# Supplementary material for: Ca2+ waves and ethylene/JA crosstalk orchestrate wound responses in Arabidopsis roots
Source: EMBO Rep. 2025 May 19;26(12):3187–203. doi: 10.1038/s44319-025-00471-z (PMC12187931; doi:10.1038/s44319-025-00471-z)
Supplement: Supplementary file 1 — Appendix [file 44319_2025_471_MOESM1_ESM.pdf]

Appendix for:

Ca^{2+} Waves and Ethylene/JA Crosstalk Orchestrate Wound Responses in Arabidopsis Roots

All correspondence: Peter Marhavý
E-mail: peter.marhavy@slu.se

Table of Contents

Title Page and Table of Contents…….Page 1

Appendix Figure S1…………………….Page 2

Appendix Figure S2…………………….Page 3

Appendix Figure S3…………………….Page 4

Appendix Figure S4…………………….Page 5

Appendix Figure S5…………………….Page 6

Appendix Figure S6…………………….Page 7

Appendix Figure S7…………………….Page 8

Appendix Figure S8…………………….Page 9

Appendix Figure S9…………………….Page 10

Appendix Figure S10…………………...Page 11

Appendix Figure S11…………………..Page 12

Appendix Figure S12…………………..Page 13

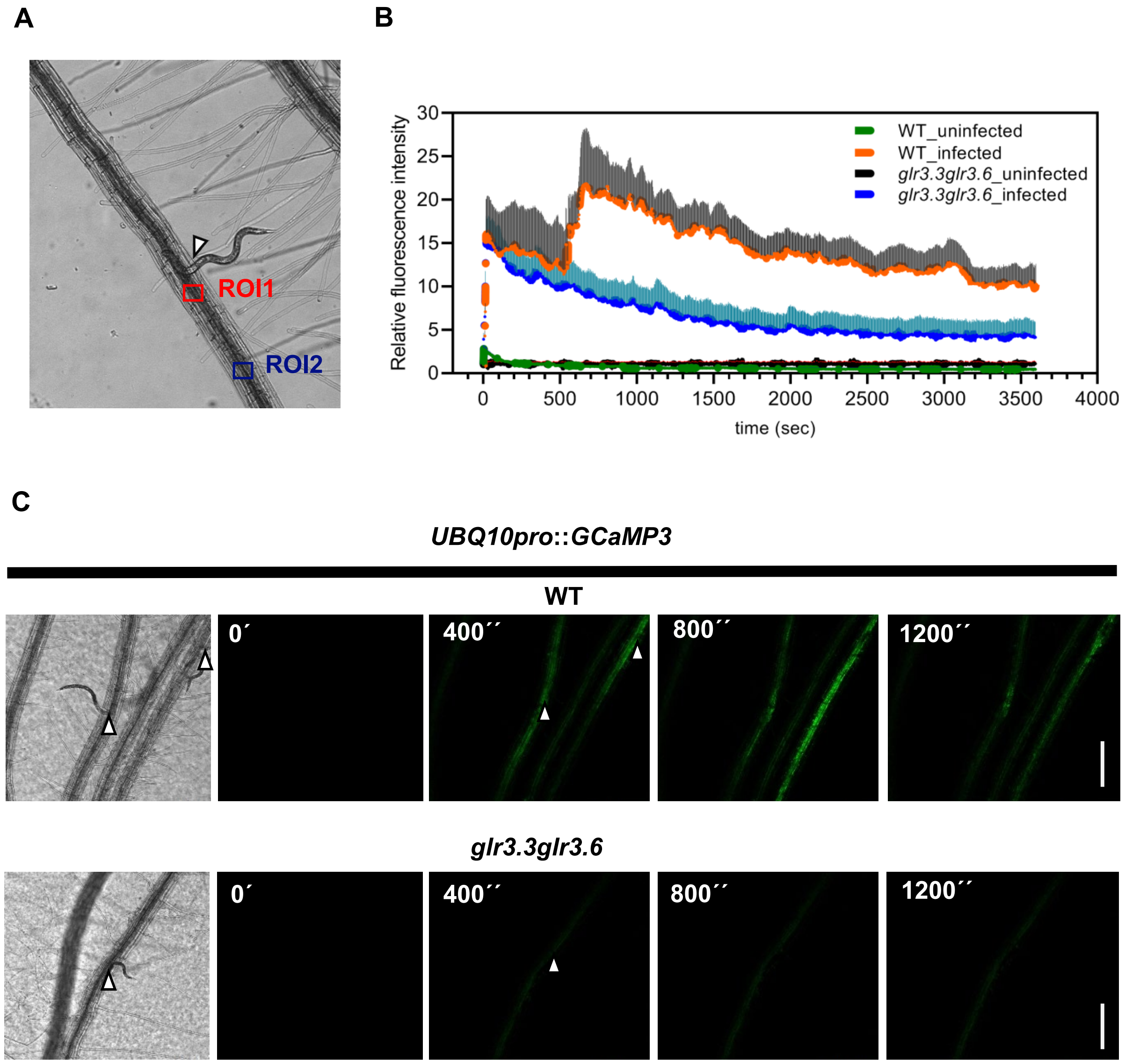

Appendix Figure S1. Nematode-induced Ca^{2+} wave in roots of Arabidopsis WT (Col-0) and *glr3.3gr3.6* using *GCaMP3* reporter lines. Related to Figure 1.

A Brightfield (BF) image (representative of several experiments) depicting the various ROI (regions of interest) selected which included both locally (ROI1) and distally infected (ROI2) regions.
B Graph depicting Ca^{2+} wave upon nematode infection in WT and the *glr3.3glr3.6* plants expressing *UBQ10pro::GCaMP3,* respectively. The data shown represents the mean ± SE of at least five independent experimental replicates. The fluorescence intensities depicted here represent the average values of the two ROIs as shown in (A).
C Representative brightfield and fluorescence images of nematode-induced Ca^{2+} wave propagation using *GCaMP3*. Time intervals are presented in seconds at the top left corner of each frame, and the white arrows represent nematode invasion points. Scale bar: 250µm.

Appendix Figure S2

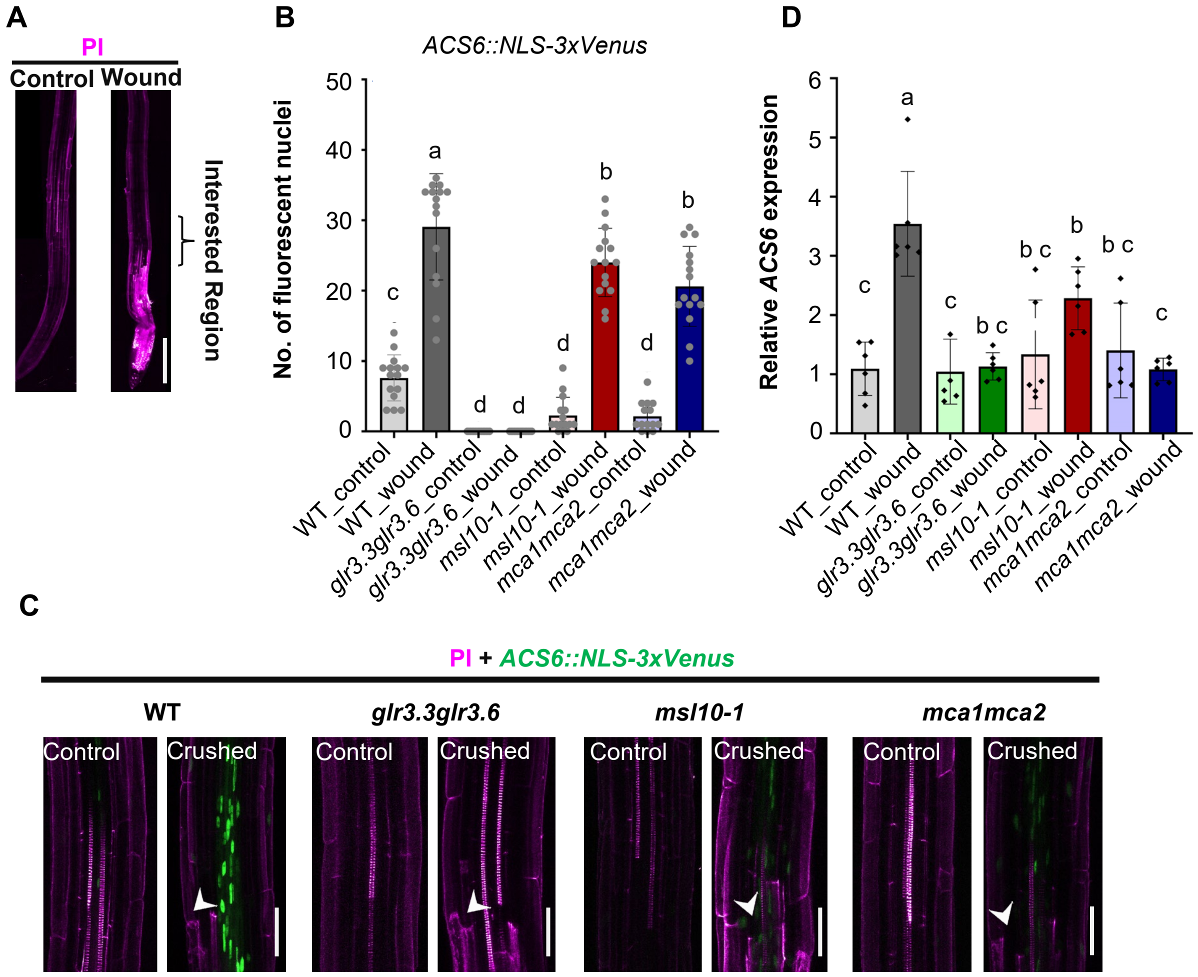

Appendix Figure S2. GLR3.3/GLR3.6, MSL10, and MCA1/MCA2 regulate *ACS6* expression upon local wounding. Related to Figure 2.

A Representative image with or without crushing the roots, the interested region for quantification as indicated. Scale bar: 200µm.

B, C Quantification (B) and representative (C) of maximum projection images of *ACS6::NLS-3xVenus* in the WT (Col-0), *glr3.3glr3.6*, *msl10-1*, and *mca1mca2*, respectively, upon crushing cells after 5 hours (control/wound). The graph demonstrates a number of cells with positive nuclear (YFP-NLS) signals. All samples showed a merged PI fluorescence and YFP channel. A white arrow indicates the position of the crushed cells region. Scale bar: 50µm. N = three biological pools, each pool includes 5 seedlings.

D Expression of *ACS6* was determined by qRT-PCR in WT (Col-0), *glr3.3glr3.6*, *msl10-1*, *mca1mca2*, with (wounding, by crushing roots) or without wounding (control). Samples were harvested 5h after wounding. N = 3 biological replicates.

Data information: In B, D, bars represent mean ± SD. Different letters (a, b, c, d) indicate statistically significant differences between groups *(P* < 0.05, one-way ANOVA followed by Tukey's test).

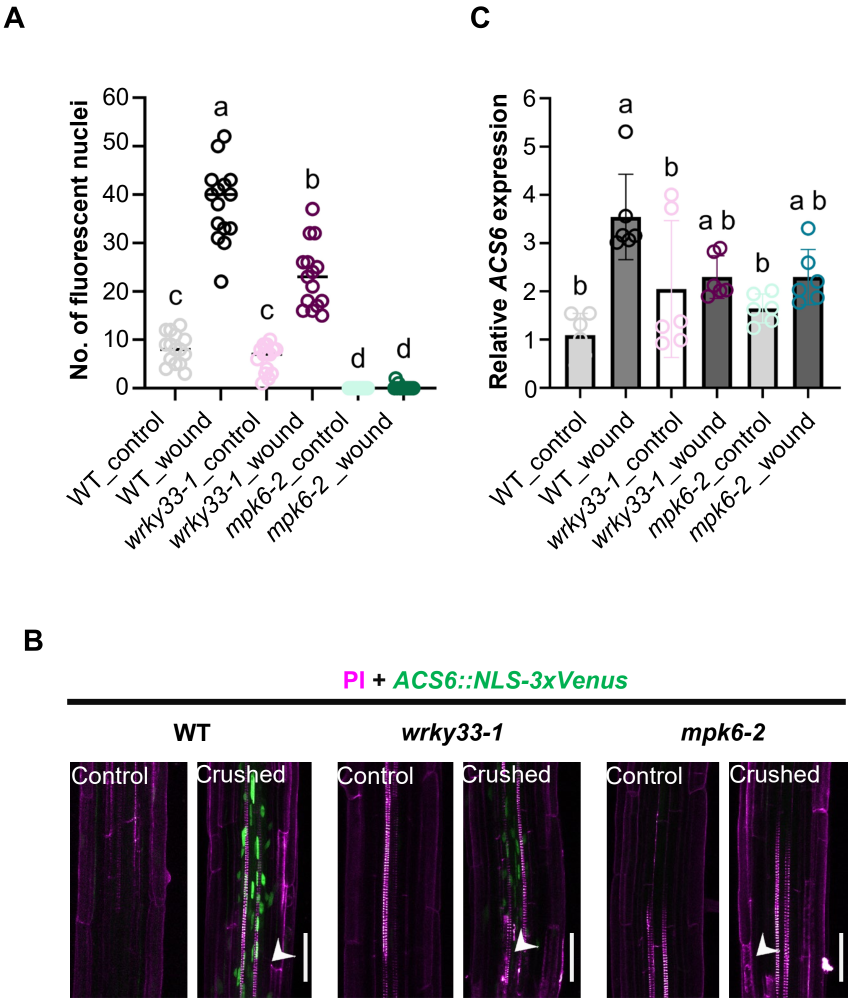

Appendix Figure S3. MPK6 and WRKY33 regulate *ACS6* expression upon local wounding. Related to Figure 2.

A, B Quantification (A) and representative (B) of maximum projection images of *ACS6::NLS-3xVenus* in the WT (Col-0), *wrky33-1, and mpk6-2*, upon crushing cells after 5 hours (control/wound). The graph demonstrates a number of cells with positive nuclear (YFP-NLS) signals. N = three biological pools, each pool includes 5 seedlings. All samples showed a merged PI fluorescence and YFP channel. A white arrow indicates the position of the crushed cells region. Scale bar: 50μm.

C Expression of *ACS6* was determined by qRT-PCR in WT (Col-0), *wrky33-1, and mpk6-2*, with (wounding, by crushing roots) or without wounding (control). Samples were harvested 5 hours after wounding. N = 3 biological replicates.

Data information: In C, bars represent mean ± SD. In A, C, different letters (a, b, c, d) indicate statistically significant differences between groups ($P < 0.05$, one-way ANOVA followed by Tukey's test).

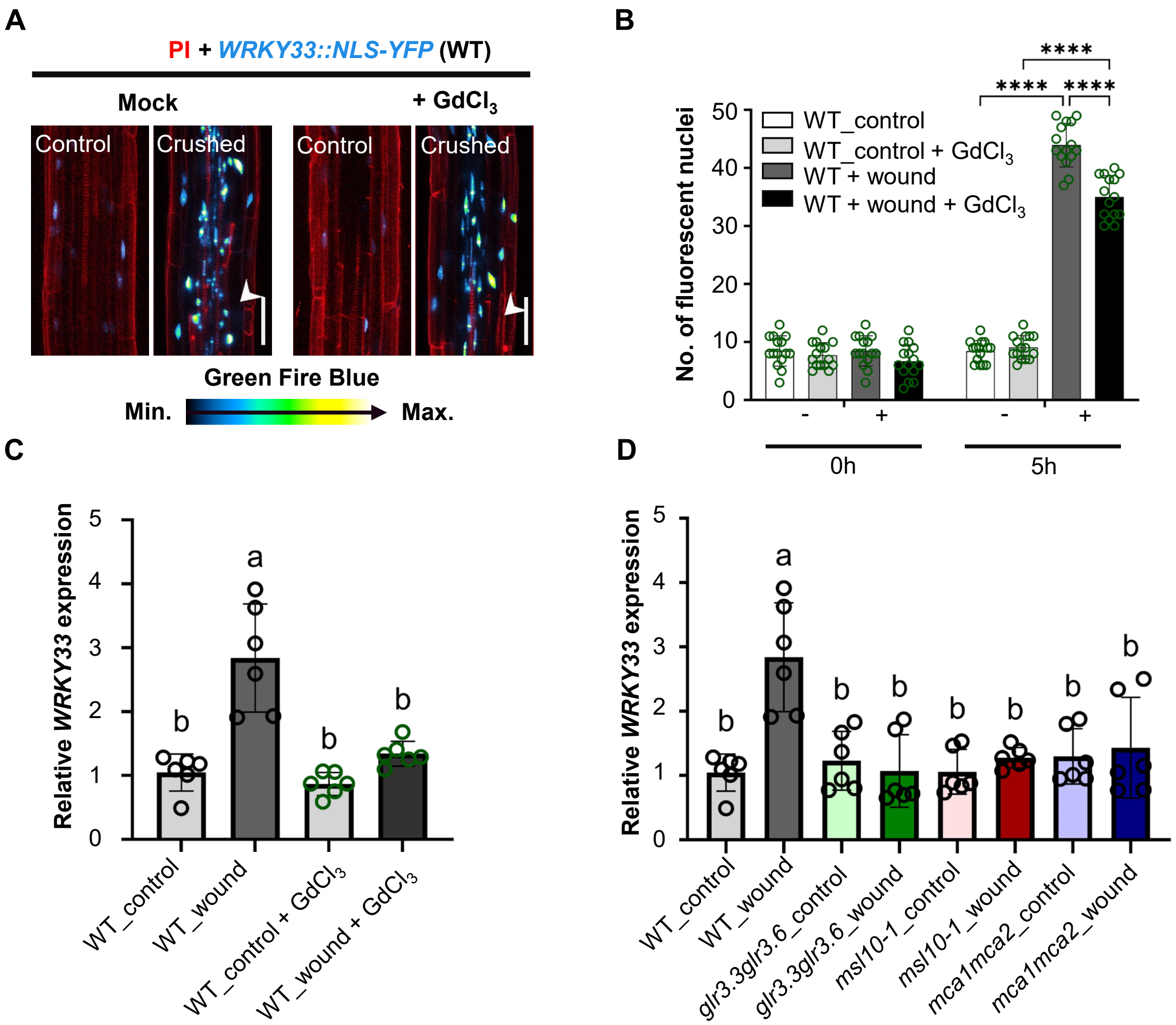

Appendix Figure S4. *WRKY33* expression upon local wounding. Related to Figure 2.
A, B Representative (A) and quantification (B) of maximum projection images of *WRKY33::YFP-NLS* in WT (Col-0), upon crushing cells after 5 hours with or without the treatment of 50µM $GdCl_3$. All samples showed a merged PI fluorescence and YFP channel. The graph shows a number of cells with positive nuclear (YFP-NLS) signals. A white arrow indicates the position of the crushed cells region, scale bar: 50µm. N = three biological pools, each pool includes 5 seedlings.
C Expression of *WRKY33* was determined by qRT-PCR in WT (Col-0) upon crushing cells after 5 hours, with or without the treatment of 50µM $GdCl_3$. N = 3 biological replicates.
D Expression of *WRKY33* was determined by qRT-PCR in WT (Col-0), *glr3.3glr3.6, msl10-1*, *mca1mca2* with (wounding) or without wounding (control). Samples were harvested 5h after wounding. N = 3 biological replicates.
Data information: In B-D, bars represent mean ± SD. In B, ANOVA Tukey's multiple comparison test was performed with a 95% confidence interval, $^{****}P < 0.0001$. In C, D, different letters (a,b) indicate statistically significant differences between groups ($P < 0.05$, one-way ANOVA followed by Tukey's test).

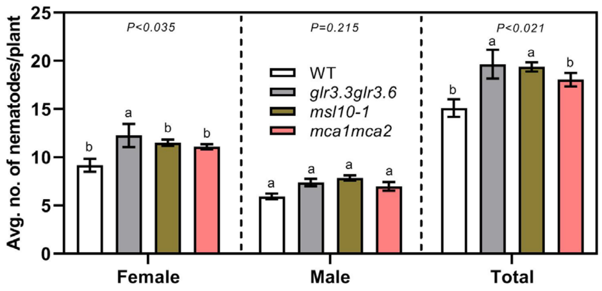

Appendix Figure S5. Nematode infection assays in WT, *glr3.3glr3.6*, *msl10-1*, *mca1mca2* plants. The figure shows the average number of nematodes per plant in Col-0 and mutant lines related to Ca^{2+} channels at 14 dpi. Bars represent the mean ± SE. The experiments were independently repeated four times, with 20–30 plants per genotype in each biological replicate. Data were analyzed using one-way ANOVA (P < 0.05), followed by Tukey's HSD post hoc test. Different letters indicate significantly different means (95% confidence).

Appendix Figure S6

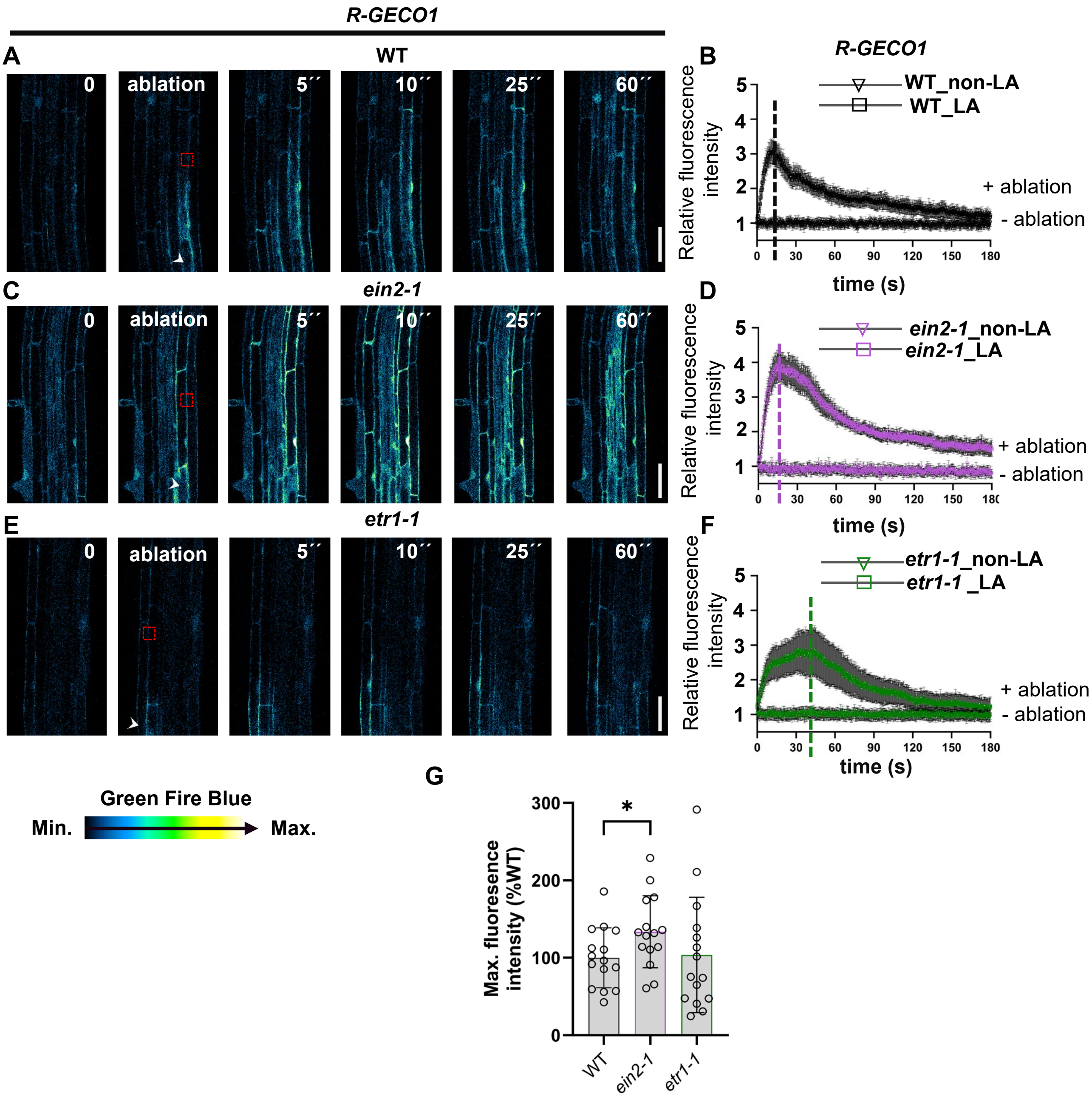

Appendix Figure S6. Ethylene pathway genes regulates Ca^{2+} wave propagation upon single-cell laser ablation. Related to Figure 3.

A-F, Representative time-lapse images (A, C, E) and quantification (B, D, F) of calcium wave propagation after cortex cell ablation in WT (Col-0) (A, B), *ein2-1* (C, D), *etr1-1* (E, F) expressing *R-GECO1*. WT (Col-0); *R-GEOC1*, *ein2-1; R-GECO1, etr 1-1; R-GECO1* (n = three biological pools, each pool includes 5 seedlings). In (A), (C), and (E), time points in seconds (s) at the top right corner of each frame, a scale bar indicates 50μm, a white arrow indicates the position of the ablated cell, and a red frame indicates a region of signal quantification. In (B), (D), and (F), error bars indicate a standard error.

G $[Ca^{2+}]_{cyt}$ peak indicated by *R-GECO1* fluorescence maximum relative intensity after laser ablation. The graph shows average $[Ca^{2+}]_{cyt}$ peaks normalized to WT. Bars represent mean ± SD. ANOVA Tukey's multiple comparison test with a 95% confidence interval and Student's *t*-test; unpaired, two-tailed, ns: not statistically significant, *$P < 0.05$.

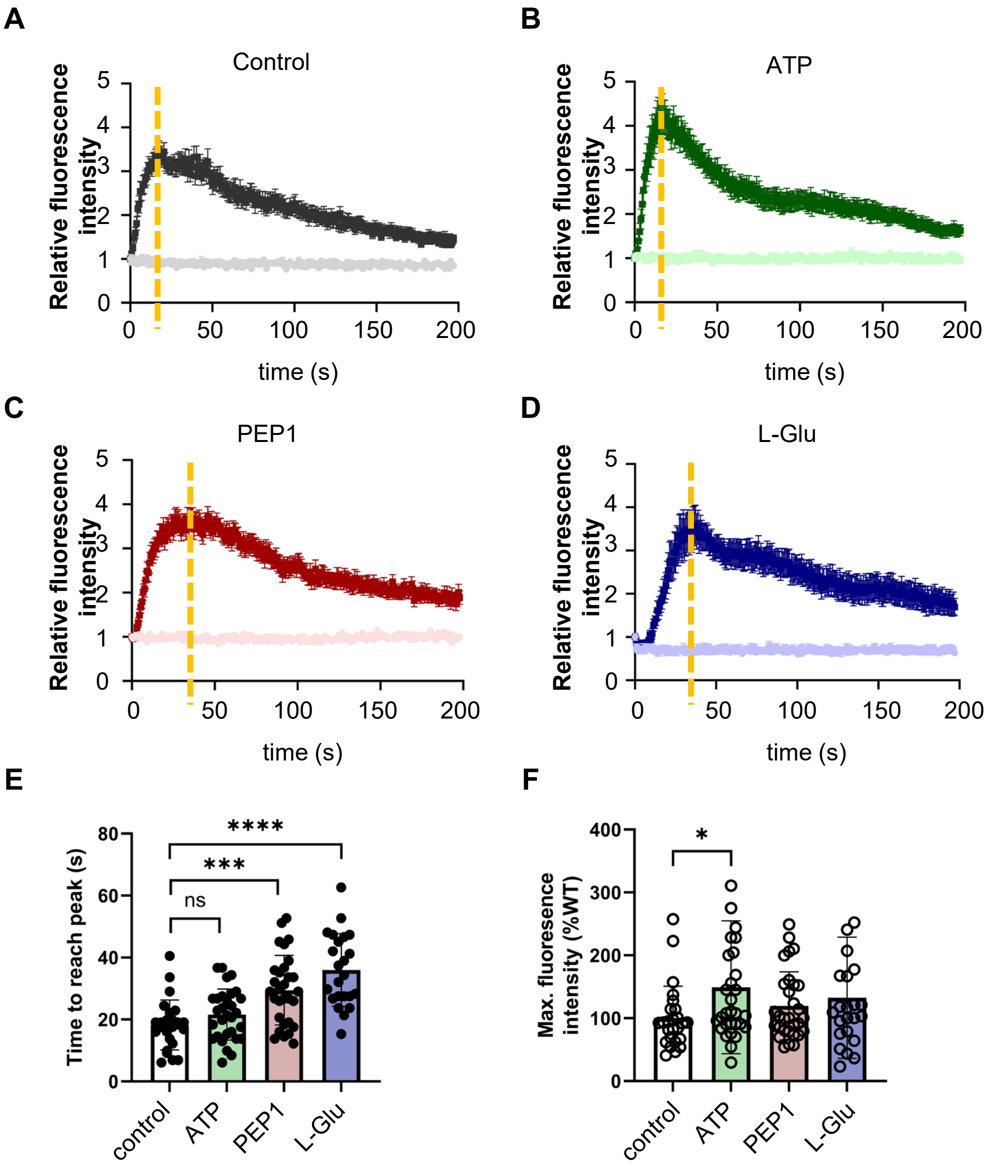

Appendix Figure S7. Applying ATP, PEP1 and L-Glu, altered Ca^{2+} wave propagation upon single-cell laser ablation. Related to Figure 3.

A-D Quantification of calcium wave propagation after cortex cell ablation using an *UBQ10pro::GCaMP3* fluorescence reporter line in WT (Col-0) under control condition (A), with 50µM eATP (B), 1µM PEP1 (C), 100µM L-glutamate (L-Glu) (D). In (A), (n = 25, three biological pools, each pool includes 7-10 seedlings). In (B, C) (n = 30, three biological pools, each pool includes 10 seedlings). In (D), (n = 23, three biological pools, each pool includes 7-10 seedlings). In A-D, error bars indicate a standard error.

E Calcium speed was quantified as the time it takes for the relative fluorescence intensity of *UBQ10pro::GCaMP3* to reach the maximum for the region (green frame indicated in Fig 1A).

F $[Ca^{2+}]_{cyt}$ peak indicated by *UBQ10pro::GCaMP3* fluorescence maximum relative intensity after laser ablation. The graph shows average $[Ca^{2+}]_{cyt}$ peaks normalized to WT.

Data information: In E, F, bars represent mean ± SD. ANOVA Tukey's multiple comparison test with a 95% confidence interval and Student's *t*-test; unpaired, two-tailed, ns: not statistically significant, *P < 0.05, ***P < 0.001, and ****P < 0.0001.

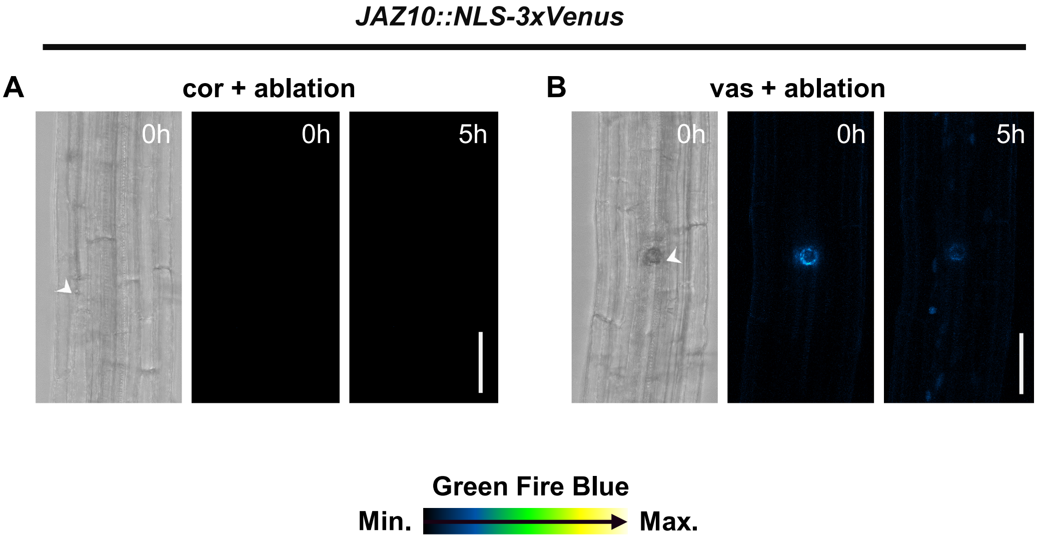

Appendix Figure S8. JA responds to single-cell ablation in a tissue-specific manner. Related to Figure 4.
A, B Representative of maximum projection images XYZ of *JAZ10::NLS-3xVenus* in WT (Col-0), at (0h) and 5 hours (5h) after laser ablation in cortex cell (cor) (A) and in the vascular region (vas) (B). Time points in hours (h) at the top right corner of each frame. A white arrow indicates the position of the ablated cell, scale bar, 50μm.

Appendix Figure S9

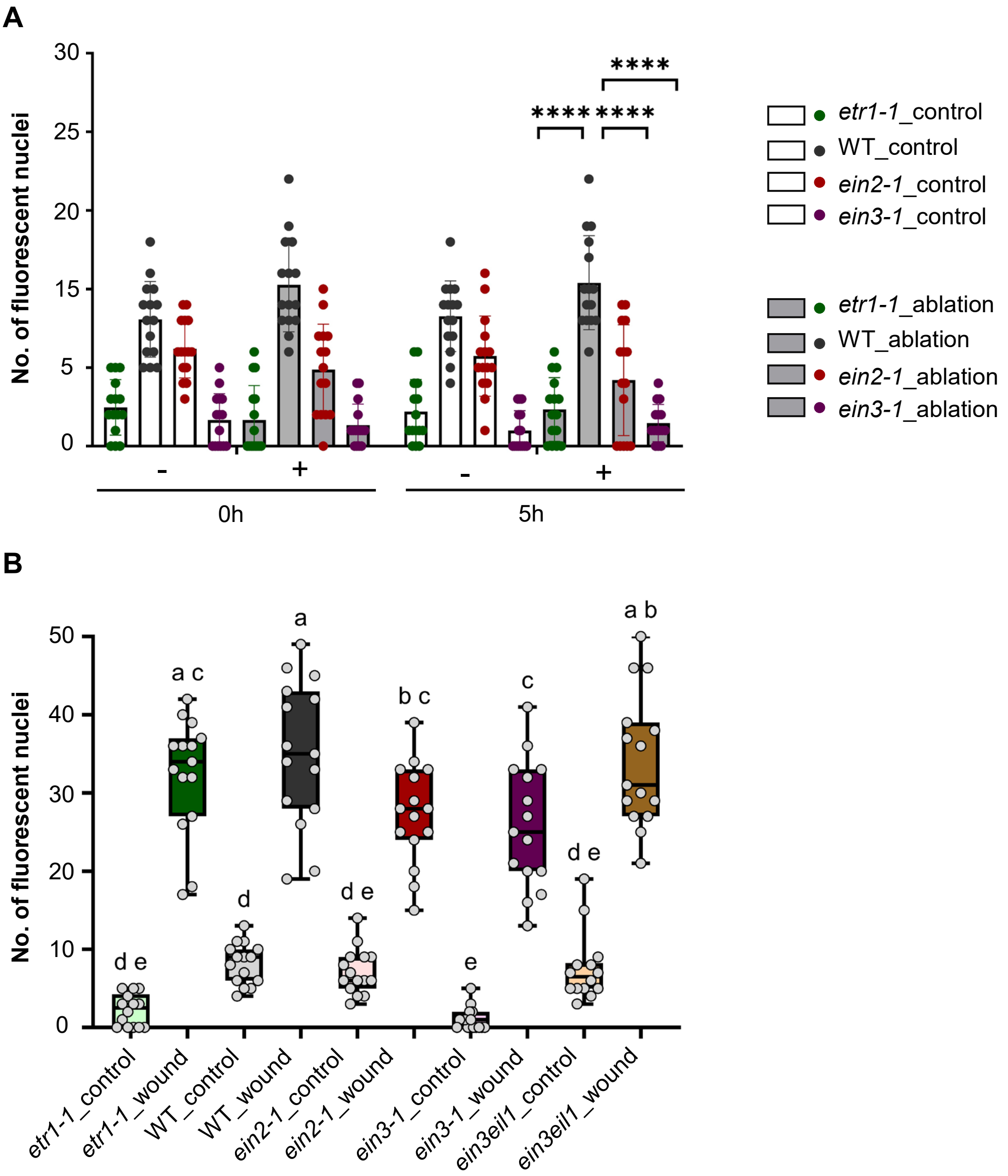

Appendix Figure S9. JA is negatively regulated by ethylene pathway genes upon local wounding. Related to Figure 4.

A Quantification maximum projection images XYZ of *AOS::NLS-3xVenus* in WT (Col-0) ethylene mutant *etr 1-1*, *ein2-1*, *ein3-1*, before (0h) or 5 hours (h) after laser ablation in the cortex cells, in a number of cells with a positive nuclear (NLS-3xVenus) signal. Bars represent mean ± SD. N = three biological pools, each pool includes 5 seedlings (ANOVA Tukey's multiple comparison test with a 95% confidence interval, and ****$P < 0.0001$).

B Quantification of representative of maximum projection images XYZ of *AOS::NLS-3xVenus* in WT (Col-0), ethylene mutant *etr1-1*, *ein2-1*, *ein3-1*, and *ein3eil1* after 5 hours of crushing cells, in a number of cells with positive nuclear (NLS-3xVenus) signal. N = three biological pools, each pool includes 5 seedlings. The box plot displays the median (the central line), the interquartile range (box boundaries), and the whiskers extending to the minimum and maximum values of all samples. Different letters (a, b, c, d) indicate statistically significant differences between groups *(P* < 0.05, one-way ANOVA followed by Tukey's test).

Appendix Figure S10

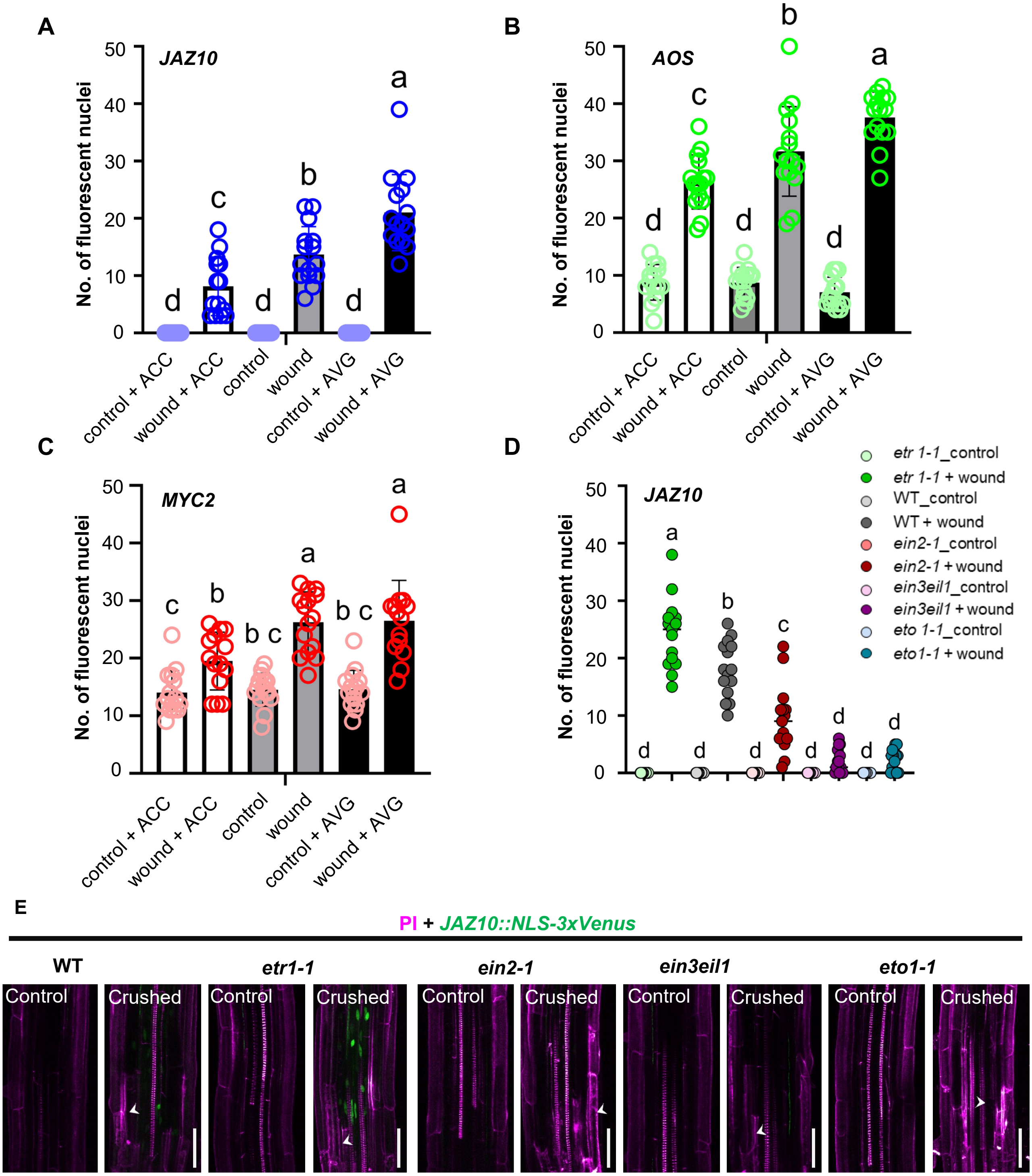

Appendix Figure S10. JA response upon local wounding is partially inhibited by ACC/ethylene pathway genes. Related to Figure 4.

A-C, Quantification of maximum projection images XYZ of *JAZ10::NLS-3xVenus (JAZ10)* (A), *AOS::NLS-3xVenus* (*AOS*) (B), *MYC2::NLS-3xVenus* (*MYC2*) (C) with or without 1µM ACC application, 1µM AVG application upon crushing cells after 5 hours, in a number of cells with positive nuclear (NLS-3xVenus) signal. D, E Quantification (D) and representative (E) of maximum projection images XYZ of *JAZ10::NLS-3xVenus* in WT (Col-0), ethylene mutant *etr1-1*, *ein2-1*, *ein3eil1*, and *eto1-1* after 5 hours of crushing cells, in a number of cells with positive nuclear (NLS-3xVenus) signal. In A-C, bars represent mean ± SD. N = three biological pools, each pool includes 4-5 seedlings. In A-D, different letters (a, b, c, d) indicate statistically significant differences between groups ($P < 0.05$, one-way ANOVA followed by Tukey's test). All images showed a merged PI fluorescence and YFP channel. A white arrow indicates the position of the crushed cells region and the scale bar indicates 50µm.

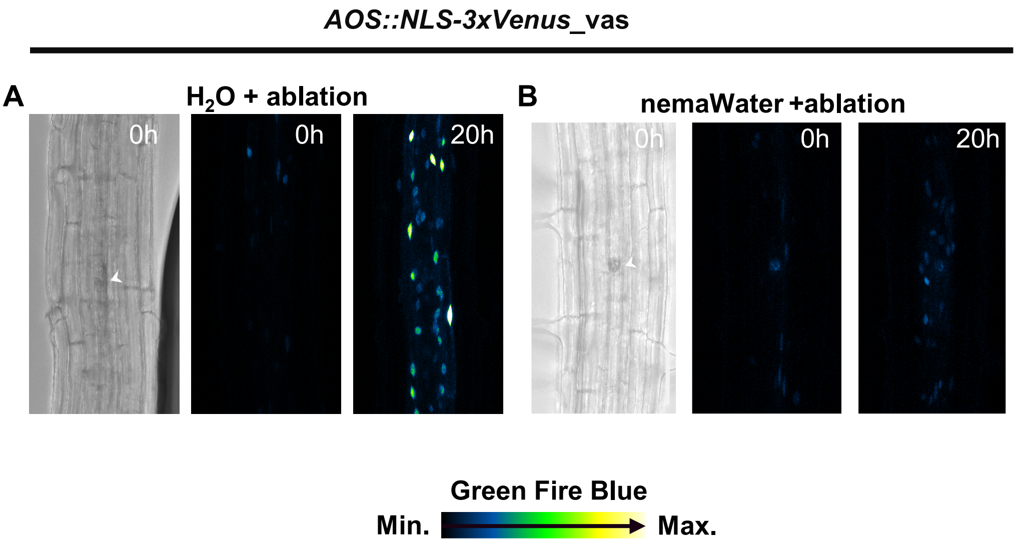

Appendix Figure S11. ***AOS*** **response upon ablation in the vascular region with nemaWater treatment. Related to Figure 4.**

A, B Representative maximum projection images XYZ of *AOS::NLS-3xVenus* in WT (Col-0), at (0h) and 20 hours (20h) after laser ablation in the vascular region (vas) with pre-treated H_2O as mock (A) or pre-treated with nematode water (B). In (A, B), seedlings were pre-treated H_2O as mock or pre-treated with nematode water for 24 hours and then performed laser ablation. Images: time points in hours (h) at the top right corner of each frame. A white arrow indicates the position of the ablated cell, scale bar, 50μm.

Appendix Figure S12

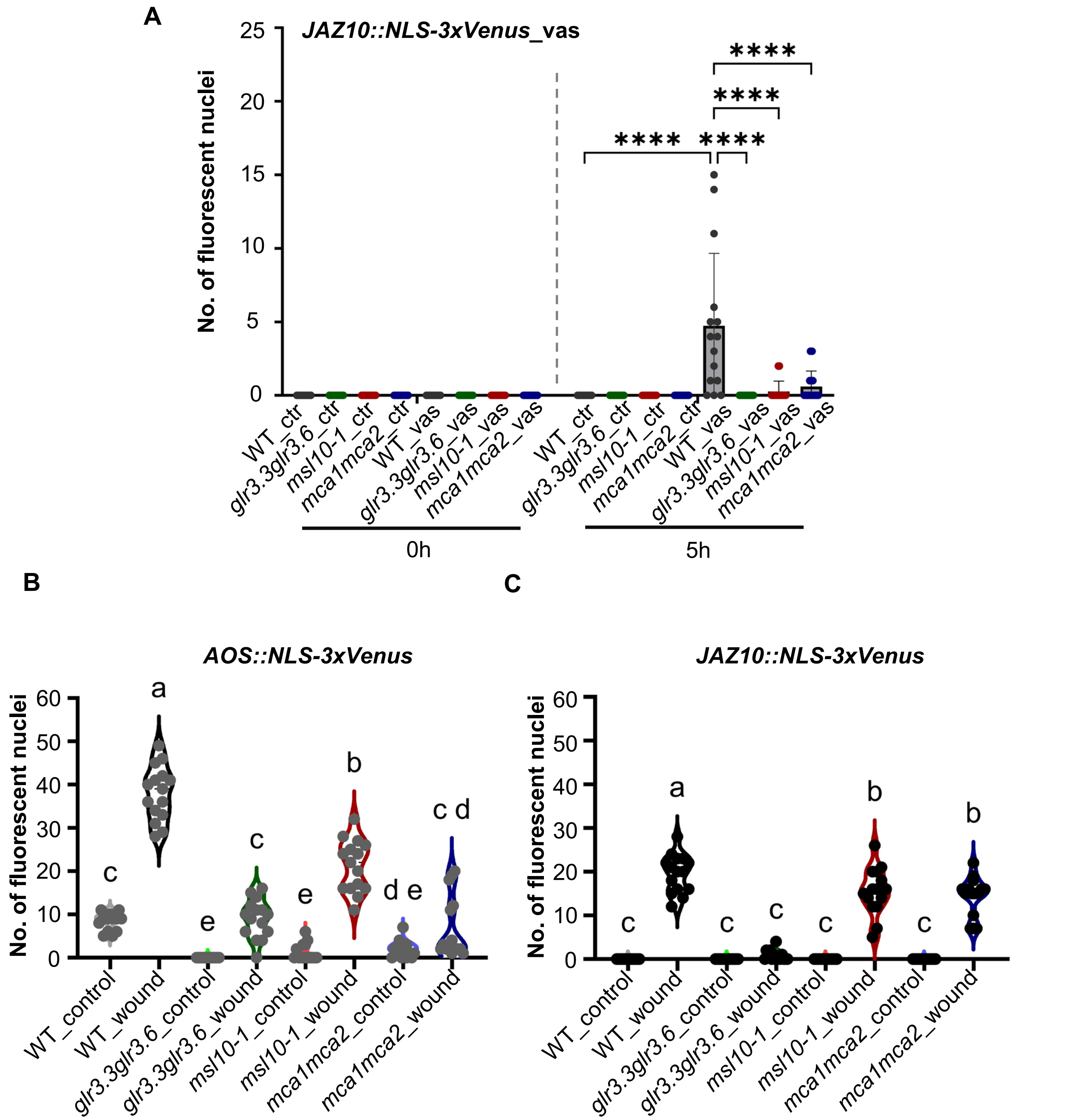

Appendix Figure S12. JA response upon local wounding is dependent on the function of GLR3.3/GLR3.6, MSL10, MCA1/MCA2. Related to Figure 4.

A Quantification of maximum projection images XYZ of *JAZ10::NLS-3xVenus* in WT (Col-0), *glr3.3glr3.6*, *msl10-1*, and *mca1mca2*, before (0h) or 5 hours (5h) with or without (control, written, ctr) laser ablation in the vascular region (Vas), in a number of cells with a positive nuclear (NLS-3xVenus) signal in all genotypes. Bars represent mean ± SD. ANOVA Tukey's multiple comparison test was performed with a 95% confidence interval, and ****$P < 0.0001$.

B, C Quantification of maximum projection images XYZ of *AOS::NLS-3xVenus* (B) and *JAZ10::NLS-3xVenus* (C) in WT (Col-0), *glr3.3glr3.6*, *msl10-1*, and *mca1mca2*, respectively, 5 hours after crushing cells, in a number of cells with a positive nuclear (NLS-3xVenus) signal in all genotypes. In (A-C), N = three biological pools, each pool includes 5 seedlings. In B, C, different letters (a, b, c, d) indicate statistically significant differences between groups ($P < 0.05$, one-way ANOVA followed by Tukey's test).
